# The Need for Psychedelic-Assisted Therapy in the Black Community and the Burdens of Its Provision

**DOI:** 10.3389/fpsyt.2021.774736

**Published:** 2022-01-20

**Authors:** Darron T. Smith, Sonya C. Faber, NiCole T. Buchanan, Dale Foster, Lilith Green

**Affiliations:** ^1^Department of Sociology, The University of Memphis, Memphis, TN, United States; ^2^Bioville GmbH, Leipzig, Germany; ^3^Department of Psychology, Michigan State University, East Lansing, MI, United States; ^4^Neuro-Source, Memphis, TN, United States

**Keywords:** African Americans, Black Americans, Black Canadian, racial trauma, race-based harassment and discrimination, psychedelic research, psychedelic-assisted therapy

## Abstract

Psychedelic medicine is an emerging field that examines entheogens, psychoactive substances that produce non-ordinary states of consciousness (NOSC). 3,4-methylenedioxymethamphetamine (MDMA) is currently in phase-3 FDA clinical trials in the United States (US) and Canada to treat the symptoms of posttraumatic stress disorder (PTSD). MDMA is used in conjunction with manualized therapy, because of its effectiveness in reducing fear-driven stimuli that contribute to trauma and anxiety symptoms. In 2017, the FDA designated MDMA as a “breakthrough therapy,” signaling that it has advantages in safety, efficacy, and compliance over available medication for the treatment of trauma-, stress-, and anxiety-related disorders such as PTSD. In the US and Canada, historical and contemporary racial mistreatment is frequently experienced by Black people via a variety of macro and micro insults. Such experiences trigger physiological responses of anxiety and fear, which are associated with chronically elevated stress hormone levels (e.g., cortisol and epinephrine), similar to levels documented among those diagnosed with an anxiety disorder. This paper will explore the benefits of entheogens within psychedelic assisted-therapy and their potential benefits in addressing the sequelae of pervasive and frequent negative race-based experiences and promoting healing and thriving among Black, Indigenous and other People of Color (BIPOC). The author(s) discuss the ethical responsibility for providing psychedelic-assisted therapy within a culturally competent provider framework and the importance of psychedelic researchers to recruit and retain BIPOC populations in research and clinical training.

## Introduction to Psychedelic Therapies

Post-traumatic stress disorder (PTSD) can be debilitating for many people, as triggers (thoughts, visuals, sounds, smells) reproduce the same physiological effect within the body as if the individual is experiencing the actual trauma again at that moment (1). The current, most successful methods of treating trauma or disorders caused by trauma can be incredibly harrowing for the patients involved, as they often require that patients retell and, thereby relive, their traumatic experiences (1). Though exposure-based therapies are the most effective for PTSD, individuals are asked to revisit traumatic events dozens of times until they become fully desensitized. These treatments often trigger extreme emotional reactions and can reactivate traumatic memories, which, in turn, may lead to a reluctance to seek professional mental health care (2). There is, however, a therapy through which patients have been able to experience past traumatic events while attenuating the anxious activation often associated with discussion of past traumas (3). The patient/client can examine their trauma and their past, explore it from different vantage points, discuss it without becoming retraumatized, process it in new ways, and eventually sever the associated misplaced feelings of guilt or shame. This is the promise of psychedelic medicine, which multiple studies have show can accelerate the healing process (4, 5).

These substances, long known in antiquity to provide therapeutic benefits, have only recently been rehabilitated and put to test in clinical studies to provide evidence for psychological distress. Psychedelic medicine is an emerging field of research, clinical and spiritual practice that examines substances classified as hallucinogens in the human mind, body, and spirit (6). Psychedelics, also referred to as entheogens, have been used for thousands of years worldwide in religious ceremonies, rituals, and healing (7). The discovery of the psychoactive properties of substances such as LSD has led to research into other classical psychedelics including mescaline and psilocybin, which are now offering a novel avenue for the treatment of patients with an array of psychiatric disorders that current medications cannot entirely help (8). However, this space has been largely limited to White people in the past 150 years for a multitude of reasons from lack of access to concerns of legality, particularly with the over-representation of black and brown inmates from drug-related offenses to the limitation in the racial makeup of research subjects and scholars (9, 10).

Throughout this paper, we will use PTSD as a model indication because there is a greater body of literature and more empirical studies addressing this indication. For people of color, however, the emotional injury of *racial trauma* is the overwhelming psychological harm for which improved tools are needed. Racial trauma, sometimes resulting in PTSD, has been neglected as a serious research subject, lacks established diagnostic criteria, and most alarmingly, suffers from a lack of clear treatment recommendations (11). Psychedelics are under observation as a useful tool for the treatment of racial trauma among a range of psychological disorders. In this paper, the authors will foreground the racial trauma specifically experienced by Black^1^ Americans, but we recognize more broadly the experiences of Black, Indigenous and people of color (BIPOC), who all share specific histories with racial oppression.

This paper will first provide a brief history of and examine the current status of psychedelic-assisted therapy as it pertains to BIPOC and the reason why these substances can be so helpful in treating race-based trauma. This paper also seeks to illuminate the multi-layered gap between the approval of these new substances and their use in BIPOC communities. Finally, the authors will discuss how this gap is being bridged and offer suggestions in improving greater access with regards to researchers and participants of color.

It is important to note that psychedelic substances are not designed to be a panacea of racial healing or trauma healing. Without a trained specialist in the treatment of trauma, and specifically race-based trauma, these experiences can leave the patient vulnerable to their emotions and further add to their trauma (e.g., (14)). In fact, true healing relies on societal change within multiple institutions and community support along with corrective interpersonal experiences of safety. When used appropriately in conjunction with integrative therapy and self-reflection processes, psychedelics can act as a powerful adjunct to psychological healing.

## Positionality Statement

The authors of this manuscript are Black and White^2^ researchers in mental health and biotechnology, with direct experience treating patients with psychedelics or research interests in psychedelics. The authors have experienced or witnessed racism that has resulted in increased stress, depression, anger, shock, and trauma. It is the position of this paper that BIPOC individuals should be able to access approved psychedelic-assisted therapy treatments (with an experienced therapist/facilitator) for emotional healing after exposure to psychological trauma and the psychobiological consequences that can follow.

The authors have interviewed numerous Black people who have suffered from racial trauma as a result of large and small wounds from a lifetime of discrimination. These individuals are in immediate need of mental health services, yet express a fear of psychedelic medicine, in particular, the vulnerability that an altered consciousness brings with it. These concerns are not unfounded. Psychedelic-assisted therapy can be unsafe when it is led by an inexperienced provider or a therapist who does not understand the nuances of racism (14). This paper aims to shine a light on the situation of those in the BIPOC community who could benefit from psychedelic treatments and provide a road map for greater access to psychedelic-assisted treatment.

## The History and Politics of Psychedelic Research

The first wave of the modern psychedelic movement in the US came in the 1950's and 1960's, at a time when young people were more openly experimenting with cannabis, heroin, and magic mushrooms (psilocybin). At the same time, scientists were studying these compounds, and clinical researchers were finding positive medicinal effects of psychedelics in the treatment of psychiatric disorders. These investigations yielded valuable information, though often from unwitting participants of color through government-funded research (15). The CIA actively studied the effects of lysergic acid diethylamide (LSD) and other mind-altering drugs to assess their use as agents of mind-control and psychological torture in over a hundred experiments under a project labeled MK Ultra (15). Often in the setting of prisons and hospitals, the limits of these substances were explored in test subjects who were not provided the courtesy of informed consent nor had the power to decline. Strauss and colleagues (15) in a recent review have documented the unjust and inhumane utilization of primarily Black racialized, incarcerated men and others institutionalized as psychiatric patients in numerous questionable experiments with shoddy and unethical methods.

Scientific research into psychedelics in the US ground to a halt in the 1970's. With ongoing political unrest over Civil Rights and the Vietnam war, the Nixon administration stoked fears of lawlessness in efforts to justify punitive action taken on this growing use into mind altering compounds. Nixon's “War on Drugs” campaign commenced in 1971 and would equate anti-Vietnam war protests along with Blacks and Latinos with newly designated illegal drugs (16). John Ehrlichman, former White House counsel and Chief Domestic Advisor under Nixon, ultimately admitted to *Harper Magazine* in 2016, “We knew we couldn't make it illegal to be either against the war or Black, but by getting the public to associate the hippies with marijuana and Blacks with heroin, and then criminalizing both heavily, we could disrupt those communities… Did we know we were lying about the drugs? Of course, we did” (17). Nixon set in motion decades of federal policies through the disproportionate arrests and incarceration of Black and Latino men for the transgression of experimenting with psychedelics and other healing substances.

The repercussions of the Nixon administration's heavy-handed federal drug policy took on a life of its own as each presidential administration has since continued policies aimed at the persecution and criminalization of racialized minorities for the use of these substances (e.g., Nancy Reagan's “Just Say No” campaign and Bill Clinton's 1994 crime bill were part of the effort to revisit and expand the War on Drugs; (16)). These unjust policies were carried forward, as it came to light later that the CIA was involved in the disbursement of drugs such as crack cocaine in the 1980's to Black neighborhoods and even profiting off the sales (18). This was all occurring despite the equivalent rate of sale and consumption in White communities (19, 20). For example, part of the Reagan-era Anti-Drug Abuse Act included a penalty known as the “100-to-1 sentencing ratio.” for the same amount of crack cocaine (typically used by Blacks) as powdered cocaine (typically used by Whites). A minimum penalty of 5 years was given for 5 grams of crack cocaine but for 500 grams of powdered cocaine, in a deliberate attempt to keep White Americans out of the dragnet (e.g., 21, 22). With the stroke of a presidential pen, Black men would find themselves in shambles, hampered with a trail of life-long negative consequences associated with having greater contact with biased law enforcement and the potential of lethal force (23).

The negative repercussions of this era remain an issue and have left stigmas in both the affected communities and the research into substances classified as illicit in the 1970's and 1980's. Psychedelic research later regained some traction in the 1990's, and its increase up to the current era has been slow. America is attempting to come full circle. The country is gradually reckoning with the racial trauma BIPOC have experienced historically at the hands of a racially biased society and its institutions (24). These traumas, occurring at the micro and macro level of society, include police violence and the explosion of the prison industrial complex sanctioned through the “War on Drugs.” Meanwhile, society is gradually recognizing that some of the very drugs at the root of the pathologic expansion of the modern prison industrial complex, which were originally intended to facilitate disproportionate incarceration of Black and Brown Americans to increase racial divisions, can instead allow for the healing of these traumas.

As this paper will highlight, after decades of scientific research with organizations such as Multidisciplinary Association for Psychedelic Studies (MAPS), the use of psychedelics as an adjunct in the treatment of mental health disorders is on the verge of gaining legal ground within the medical community. Communities of color have notoriously been and are still left out of these spaces. The authors intend to illuminate this disparity and offer ideas to bridge the gap so that BIPOC can benefit from these resources and treatment opportunities as one way to help heal from the ills brought on by societal oppression.

## The Persistence of Anti-Black Racism as a Precursor to PTSD

Although many minoritized persons in North America experience discrimination based on their ethnicity, anti-Black sentiment remains one of the most pervasive types of negative stereotyping with significant and well-documented health impacts (25). Being Black in the US still means learning to live with the persistent threat of race-based stress in the form of slights, slurs, insults, violence and even death (25). A single negative racialized event can certainly trigger post-traumatic stress disorder, but the cumulative effects of racial trauma in the Black community are often the catalyst for a transformative traumatic event.

The DSM-5 defines PTSD as first having been exposed (directly or indirectly) to trauma, followed by symptoms from that exposure causing disruption in one's life in a multitude of ways. Criterion A specifically defines trauma as direct or indirect exposure to or threat of an experience that includes death, serious injury or sexual violence (26, p. 271). The indirect exposure includes witnessing an event, learning of a loved one's event or being exposed to “aversive details” of an event (26, p. 271). With these new guidelines, the DSM-5 provisions are more inclusive of the detrimental effects of modern forms of Black antipathy and bias than the previous fourth edition manual. This expanded definition of PTSD encompasses what clinicians already see—African Americans live with higher rates of PTSD, as reported in the scientific literature (27).

Racial events include a direct physical assault, a traumatic event (or trauma-inducing experiences) involving close family members as well as individuals frequently exposed to graphic details about trauma (28). Racially traumatizing events may comprise experiences such as relentless microaggressions, police profiling, and also systemic racism in law enforcement, healthcare and education. Black people throughout the US and Canada experience some form of racial microaggression as a near daily occurrence from the micro to the macro levels of society, including cultural and historical traumas (29). According to Sue and colleagues (30), “Almost all interracial encounters are prone to microaggressions.” Racial mistreatment or microaggressions are subtle acts of dehumanization that can range from poor service in restaurants and unsolicited hair touching to race-based doxxing or swatting, verbal insults and outright physical violence (25, 31, 32). These can be considered racially traumatizing experiences as research shows that the severity and prevalence of PTSD symptoms do not change as a function of if the experience meets Criterion A (33–35) as well as findings that demonstrate that such events are connected to PTSD symptoms above and beyond Criterion A (36).

Black communities are mentally and emotionally impacted by public displays of inhumanity (e.g., the epidemic of extrajudicial police killings; 37–39), as they routinely witness racialized tragedies committed against people who look similar to them and inhabit the same social circles. Part of the cycle of trauma for Black people is the discounting or outright denial of the injury's existence and one's racialized reality by other citizens who do not experience anti-black sentiment (40). With enough frequency and unpredictability, such racial stressors can lead to racial trauma, and the accumulation of these negative occurrences can have real long-lasting consequences for Black mental health ((41–43)).

## The Physiological and Psychological Effects of Race-Based PTSD

In an extensive review of the causes of race-based trauma linked to PTSD in communities of color, Williams and colleagues (24) described racial trauma as a cumulative psychological injury caused by hate or fear of an individual due to their ethnicity or race that overwhelms the individual's ability to cope. Racial trauma is associated with severe physiological and psychological harm across BIPOC populations (44). The harm is especially pernicious because it is associated with an immutable characteristic or identity (e.g., one's race) which can cause the consequences to further worsen over time, resulting in symptoms of PTSD and a variety of other psychopathologies (24, 44). Notably, traumatic experiences and PTSD can initiate a cascade of consequent psychological, physiological and epigenetic effects on the body (45).

Ongoing racial dehumanization weathers the brain, mind, and body, leaving deep intergenerational scars at the molecular level (46). Those affected by racial trauma frequently report both psychological and physical symptoms that are associated with PTSD including trauma-related emotional arousal and reactivity (e.g., hypervigilance, irritability), labile mood, intrusive thoughts, exaggerated startle response, flashbacks, and sleep disturbances, such as insomnia and nightmares (47, 48). These symptoms are correlated with abnormal hormone and neurotransmitter levels (e.g., serotonin, GABA, dopamine, cortisol) as well as abnormal brain wave morphology (49). Such dysregulation of the brain and body leads to poor physical and psychological health outcomes (46, 50, 51).

Physiologically, the presence of ongoing arousal from daily systemic racism triggers a stress response within a feedback loop system known as the hypothalamic pituitary adrenal axis (HPA-axis), which is responsible for the proliferation of cortisol, epinephrine (i.e., adrenaline) and norepinephrine (52). This system regulates the body's fight, flight, or freeze response, which is often associated with increased heart rate, shallow breathing and fatigue. This process can be activated in as little as a moment of real or imagined threatening stress (53) and can leave the body susceptible to illness and disease, particularly when experienced frequently over time (54). Correspondingly, those subjected to racialized trauma have persistently elevated stress hormone (e.g., cortisol) levels, which is associated with gastrointestinal distress, hypertension, diabetes, heart disease, stroke, and an overall lowered immune response (38, 52).

Just as the body reacts poorly under chronic stress, the brain's stress response causes a disruption of neurotransmitters, neural networks and brain communication and signaling (55). Brain imaging of neurons has demonstrated how dendrites—tiny projections of branches at the terminal end of neurons— shrink in a stressful social environment (56). Studies utilizing quantitative electroencephalography (qEEG) also exhibited dysregulation of brainwave frequency homeostasis in similar circumstances (57–59). Brain frequencies function within a very narrow oscillatory window (60). If they oscillate in frequency or amplitude outside this normal range, it can signal pathology within the brain and mind (61). These frequencies can become dysregulated as a result of childhood trauma—neglect, emotional abuse, sexual abuse, food scarcity, lack of bond with primary care giver (i.e., birth mother), incarcerated family member—creating heightened vulnerability to later psychiatric illness (62). Trauma has numerous effects within the cortical framework, interfering with an individual's capacity to feel, think, and connect with others. Notably, many diagnosed with a trauma-related disorder describe a lack of pleasure and a disassociation from those around them including feelings of depression, difficulties with planning, thinking, and memory (63, 64).

Historically, psychiatry has underutilized the neuroscience of the brain and the medical community has been slow to utilize the newest brain science (65). Furthermore, as has been reported in other areas of medicine, there are disparities is the quality of care patients receive, with Black patients being less likely to receive psychiatric treatment referrals or to be diagnosed with PTSD by a licensed psychiatrist or psychologist, and PTSD secondary to racial trauma is under diagnosed and under treated (63, 66, 67). Raced-based PTSD results in profound physiological and psychological damage (63, 64, 68) that can cause permanent changes in the brain. Fortunately, the brain is plastic and amenable to change with effective treatment. Neurotransmitters, brainwaves and brain connectivity have been shown to normalize with utilization of neurotechnology devices and psychedelic medicines in conjunction with psychotherapy (61). With these treatments, patients have reported reduced symptomatology, limited side effects and an overall improvement in health (61). In concert, adaptive behavior, positive emotional states, and supportive relationships with others become more sustainable.

## The Potential of Psychedelics: Novel Treatments and Studies on the Horizon for Racial Trauma

Despite the clear evidence of ongoing emotional harm to Black Americans caused by systemic racism, there has been relatively little research focused on interventions (11). Psychedelics are one avenue worth exploring for their potential to help alleviate racial trauma (69). MDMA-assisted psychotherapy has already been shown to be a highly effective approach for PTSD, with three administrations of MDMA substantially more effective than traditional pharmacologic treatment methods such as life-long prescription of SSRI medication (70, 71). As such, MDMA-assisted therapy was designated a “breakthrough therapy” by the FDA in 2017 and is now being made available through the Expanded Access (compassionate use) program.

Williams and colleagues (72) found significant reductions in depression, anxiety, and stress following naturalistic use of psychedelics for racial trauma. These results are consistent with the increasing evidence that psychedelic substances can catalyze healing for those suffering from various mental disorders (3). With the breakthrough designation of MDMA and psilocybin by the FDA for PTSD and depression (see Table 1) and the publication of at least 14 well-designed clinical studies on LSD, MDMA, psilocybin and ayahuasca for a range of mood disorders, the potential for hallucinogens as therapeutics is now being realized (73–75).

**Table 1:** Common psychedelic substances and research.

**Class**	**Common name**	**Clinical trials**	**Mechanism of Action**	**Therapeutic potential**
Psychedelics	LSD	I, II, Observational	serotonergic agonist	Alcoholism
	Psilocybin	Breakthrough Therapy Designation	serotonergic agonist	depression disorders
	Mescaline	I, II	serotonergic agonist	alcoholism, depression
	Ayahuasca (DMT)	I, II	serotonergic agonist	depression
Entactogens	MDMA	I, II, III, Breakthrough Therapy Designation	monoamine releasers and reuptake inhibitors	PTSD, Anxiety
Dissociatives	Ketamine		glutamatergic NMDA antagonists	Depression
Atypical	THC		CB1 receptor agonist	Anti-nausea, neuropathic pain, spasticity
	Cannabidiol (CBD)	I, II, III	serotonin 5-HT1A receptor partial agonist, TRPV1, GPR55 receptor modifier	epilepsy, anti-anxiety

*LSD, lysergic acid diethylamide; MDMA, 3,4-methylenedioxy-methamphetamine; NMDA, N-methyl-D-aspartate; CB1, cannabinoid 1; THC, Δ9-tetrahydrocannabinol; 5HT, Serotonin; TRPV1, Transient Receptor Potential Vanilloid; GPR55, G-protein receptor 55; DMT, N, N Dimethyltryptamine*.

Other research in this area includes ketamine-assisted therapy. Halstead and colleagues (76) provided intensive outpatient treatment for a client with treatment-resistant PTSD due to racial trauma and childhood sexual abuse. During the 13-day therapeutic intervention, clinicians administered ketamine on four occasions, integrated with mindfulness-based cognitive therapy and functional analytic psychotherapy (FAP). The treatment providers were therapists of color, using a culturally informed approach and anti-oppression lens to conceptualize the client's trauma. This case study found significant reductions in symptoms post-treatment and sustained benefits 4 months after (76).

Under current development is an open-label proposal to MAPS for a comparative effectiveness study that explores the potential enhancement of neurofeedback therapy with MDMA treatment for African Americans in the deep South with race-based trauma. If funded, this study would be the first to examine the effectiveness of entheogens and neurofeedback therapy in concert as a treatment protocol. One of the authors on this paper, an interdisciplinary scholar, researcher and licensed physician associate in mental health, has proposed a prospective single-site, randomized, controlled two-arm, between-subject comparison open-label study assessing the safety and effectiveness of MDMA-assisted therapy in combination with neurofeedback therapy in participants diagnosed with PTSD whose index trauma is a racialized event.^3^

## Racial Disparity in Diagnosis and Treatment is Multi-Layered

Once psychedelics become legal medicines, there are still grave concerns that they will remain out of reach to Black Americans. Black researchers have supported the use of entheogens such as MDMA for the healing of racial wounds with the critical caveat that providers must be well-equipped to work with clients of color or risk doing more harm than good (14). "As with much of medicine, many providers have not received in-depth and ongoing culturally sensitive training (79, 80). In fact, many providers harbor implicit biases themselves that have not been explored, to the detriment of their patients (e.g., (81–83)) and yet, they are also the gatekeepers to diagnosis and treatment. Numerous studies show that treatment and prescribing habits can differ based on patient race (84–88). Racial group affiliation is particularly influential when it comes to the diagnosis and management of mental health disorders. When presented with the same symptoms, physicians were more likely to diagnose Black patients with schizophrenia or bipolar disease while White patients were diagnosed with major depression (66, 89). The data is consistent with other studies and suggest that disparities in the diagnosis of schizophrenia result in part from clinicians misperceiving the relevance of mood symptoms among Blacks compared with other racial or ethnic groups. In addition, African American subjects have also been shown to be less likely to receive the most effective antipsychotic medications (89). Psychotropic prescription-writing habits of practitioners have been demonstrated to be significantly influenced by the provider's perception of the patient's emotional health only when it comes to their White patients and not in their Black patients. In all, these misperceptions lead to misdiagnosis, which in turn, impact treatment and prescribing patterns and lead to lower standards of care (90–93).

Specifically in the case of PTSD, publications show race disparities in the treatment and prescriptions of pharmacotherapy (94–96). For example, a racial hierarchy of treatment has been found in US veterans. Compared with White veterans, Latino veterans were less likely to receive a minimal trial of pharmacotherapy (96). Furthermore, African American veterans were the least likely to receive *any treatment at all* in the 6 months post diagnosis. This disparity in treatment of veterans with PTSD is a well-documented, significant, and ongoing issue that has been further demonstrated in a recent analysis of more than 1,500,000 veterans with PTSD (96). Despite being diagnosed with PTSD at similar rates across categories, females and Black veterans were less likely to receive PTSD disability awards, which hindered their ability to even begin treatment (97).^4^ It should be noted that the US Department of Veterans Affairs, who serve as the primary mental healthcare provider for many in the BIPOC community, are beholden to Federal restrictions on the use of psychedelics in treatment (98, 99).

The common and ongoing refrain in these studies is a lack of understanding of the emotional state of Black people, which results in habitual overdiagnosis with psychotic disorders, an underdiagnosis of mood and anxiety disorders, and simultaneous undertreatment in all areas of medicine (66, 88). Moreover, Black physicians represent only 2% of all US psychiatrists according to the American Psychiatric Association, revealing a dearth of representation (100). This further underscores the need for culturally informed clinicians training (101, pp. 37-42). What's more, there have been no manualized protocols or clinical trials for people of color with race-based trauma in psychedelic research. Given these existing disparities, concerns remain as to whether psychedelics will be appropriately provided to people of color.

In treating patients with alternative, plant-based medicines, it is also important to recognize receptiveness as a potential barrier to treatment within Black communities. Statistics show that Blacks in the US have the lowest use of any racial group for psychedelics. In addition, the starting age for psychedelics use is older than for all other racial groups (102). One explanation is simply that Blacks have been socialized to avoid psychedelics due to the disproportionate consequences of illicit use (9, 102). For instance, though arrests have dramatically dropped in states that have legalized cannabis, incarceration and life-consequences for use of mind-altering substances remain disproportionately high for communities of color in many states and counties in the US (103). In addition, Black individuals may, for good reasons, not perceive psychedelics as safe (9). The cultural stigma against psychedelic use makes it more difficult to convince Black individuals that psychedelic-assisted therapy could be a beneficial intervention. The *destigmatization* of psychedelics as a category remains an ongoing process, but the destigmatization of *Blacks using psychotherapeutics* remains further out of reach (74). Legitimizing psychedelics in the eyes of Black Americans will necessarily be a process in which those who have experienced successful therapy can convey the benefits to those suffering from treatable psychological trauma (104). A parallel societal change must also occur in which law enforcement, psychology and medicine becomes accepting and encouraging of seeing Black people as using these substances and experiencing healing from trauma. This may be a difficult road to walk, but for many seeking relief, it will be well worth the travel.

## The Challenges in Equipping Mental Healthcare Providers with Culturally Sensitive Training for Race-Based Trauma in the Use of Psychedelic Therapy

Clinical trials testing the safety and effectiveness of psychedelic-assisted psychotherapy use a protocol in which a psychedelic compound is combined with a defined sequence of therapy sessions with the goal of molding and supporting the patient's psychedelic experience. These protocols have developed into a standardized practice which, following FDA approval, will be legally required by governmental authorities every time psychedelic-assisted therapy is initiated. Use of psychedelics outside of this framework would, therefore, not be clinically or ethically acceptable. The types of sessions which are required to prepare patients, engender trust, and successfully establish a therapeutic relationship with the guiding facilitator include *preparatory, medication*, and *integration* sessions (105). The clinical facilitator's role is ultimately to assist in translating the experience into a therapeutic shift in cognition and behavior.

In order to best support patients in their future psychedelic-assisted therapy, MAPS was granted FDA approval to initiate a clinical study in 2010 where future potential providers enrolled in the volunteer trial for MDMA were permitted, according to the protocol, to receive a single dose of MDMA in conjunction with psychotherapy exactly as defined for patients in the pivotal phase III MDMA trial for patients. This step was an unprecedented but necessary step to give credibility to the clinicians and researchers who work in the psychedelic field, given the difficulty of legally organizing these types of experiences (106). The trial was designed to give providers this experience while collecting data on healthy volunteers.

Recent accounts of the experiences of BIPOC providers who have taken part in these early volunteer studies have now been published and highlight the potential as well as the challenges that other BIPOC participants may face in such trials ((14, 107)). In his psychedelic experience, a researcher who identifies as an Asian, queer male, described having vivid visions of hybrid animals and plants related to his cultural heritage, his sexual identity, and the intersectionality between the two (108). Insights gained from the integration of these experiences included proactive strategies to counter internalized racism, and the realization that he must radically accept the intersectionality of his sexual and cultural identity to overcome societal barriers of discrimination and internalized minority stress. It is important to note that the co-therapists in this study were a female of color and a White male. The author specifically highlighted the role of the therapist of color regarding his positive experience. He explained, “There was also an indescribable experience of being “seen” and “heard” as a queer person of color in a White heteronormative society simply by having an older female therapist of color siting patiently across the room, conveying acceptance, nurturance, and in a sense, non-judgmental “permission” to open up about my issues” ((108), p. 63; Buchanan, 2020).

In a differing account during the MAPS-sponsored MDMA trial, a Black female Marriage and Family Therapist describes her experience as a participant in an MDMA session. In this session, she reported initial feelings of freedom and ancestral connection, feeling the presence of her grandmother guiding her toward peace. These feelings transitioned to feelings of confusion, as racial wounds rose to the surface of her experience. When sharing her frustration with her sitting therapists, their lack of empathy led to a fundamental misinterpretation of her emotional state. Ultimately, she was received with a microaggression that deepened the disconnection between her mind and body and left her feeling grievously misunderstood. She stated that the MDMA would not allow her to isolate herself into her usual coping mechanisms and, instead, left her vulnerable to others. She suffered for many weeks from the trauma of the experience. Despite this, after much work to integrate her MDMA experience, two truths were made clear to her, “more Black folx deserve to feel human, free from the oppression and traumas we've endured, and that nothing can separate me from Divine Love” (14).

Buchanan (109) discusses these narratives and their connection to the larger Black community, and she is deeply critical of the preparation and training current psychedelic-assisted therapists and trainees are receiving to meet the needs of BIPOC populations. Facilitators first must unpack their own biases before they can then aid others in unpacking their traumas. Buchanan further notes that these therapy paradigms have not centered the needs of BIPOC people seeking healing and, hence, puts them at additional risk of harm. The participants are in a position of increased vulnerability given both the heart-opening effects of the entheogens, which strip people of their typical psychological protections used to navigate interracial interactions, and the fact that participants cannot exercise their right to leave because of the effects of the psychedelic substances. This should increase the requirements, Buchanan argues, for intersectional cultural humility (110, 111), deep and prolonged engagement in the providers' personal work on their social identities and connections to privilege and oppression (112), and understanding of identity-related factors, such as race, that influence set and setting (113).

Multiple studies have shown that people exhibit greater empathic resonance to individuals with a similar skin color (91, 114–116). The tendency to favor in-groups is so strong that the categorization of people into in-groups based on even temporary and arbitrary traits creates biases resulting in favoritism (117). In regard to White people's perception of Black people's pain, brain imaging reveals an anti-Black racial bias, (i.e. Black participant's pain was assessed less painful than White people's pain; (114, 118)). This kind of assessment of bias is compelling because it does not rely on the self-assessment of the participant, rather the mere viewing of an individual in pain causes a measurable sensorimotor resonance dependent on the perceived racial similarity between the victim and the observer (119). These studies provide a mechanism to help explain the reduction in empathy observed between Black patients and their White providers, and explain both the misinterpretations of mood symptoms in the diagnosis of schizophrenia and the disconnect in MDMA therapy, as described above (14, 66, 89).

The lack of empathy toward Black (91) and other BIPOC individuals, based on out-group bias can be mirrored in the client-therapist relationship and cannot be expected to be bridged simply by psychedelics alone (also see **Table 3** for further resources). Even in the absence of potent empathy-expanding compounds, clinicians of every background must hold up the mirror to consider if they have the cultural competence to offer therapy in a way that will not harm their client. This is not to imply that people only ever have empathy for their own in-group and that this is immutable. Research also suggests that racially-based sensitivity to others' pain can move from implicit to explicit if made salient (115). This underlines the importance of shining a light on these implicit racial disparities in the social and medical sciences. The current situation is that in the USA, most therapists are White, and most clients of color will be seeing a White therapist, this makes implicit bias an important topic. If therapists cannot not perceive the harm created by racial trauma, because they underestimate the effects of implicit bias or “colorblindness,” they will be unable to carry out this work, regardless of the manner in which it is carried out. We all see race (115, 119, 120) the question that remains is *how* we see it and what we do with this knowledge.

## Challenges Around Inclusive Training: A Post-hoc Case Study

As underscored by the previously mentioned moving accounts of psychedelic-assisted therapy, culturally relevant therapy is necessary for success (121). Ethically, only culturally competent practitioners should administer psychedelic compounds, particularly to Black patients who have inevitably experienced some racial trauma by virtue of living in racialized societies. Otherwise, there is a significant risk of causing harm to the patient, and the field of study is left vulnerable to critique (14). In one initiative to address this problem, MAPS conducted a grant-funded conference and training to teach BIPOC how to carry out MDMA-assisted therapy for PTSD (122, 123). The idea being that BIPOC providers would have greater cultural competency through life experiences on how to treat those in their communities (124). Although the conference portion, which was organized by people of color, was well-received, the training portion of the meeting, as noted by MAPS themselves, was problematic (122). We think that it is important to touch specifically on some of the reasons for the difficulties of this training because elements of the issues that emerged in the meeting regularly plague initiatives that revolve around issues of race (e.g., 125), and if we do not ask and understand the reasons, there will be that much more difficulty in providing the future training required for psychedelic-assisted therapy.

A *post-hoc* analysis of the training provides an opportunity for improvement at multiple levels. Most prominently, in the execution of the training event, there was a lack of power sharing with knowledgeable people of color. The lead trainer, appointed by MAPS, was a White woman with no experience providing diversity education (122). In training such as these, strong emotions are expected because the trainers are breaking taboos around speaking about the intersection between race and identity and the role of the individual in an unjust society (125, 126). An issue which often emerges is the difference in empathy and perception of racial issues between the White participants and those of color (e.g., 113). Many White Americans are too often concerned with denials of BIPOC experiences and defensive strategies to avoid the hard truths of everyday racism (126). Others attempt to skip to uncritical, poorly thought-out solutions to address complex, systemic issues on the nation's racist past and contemporary realities. Due to this, White participants often require lengthy, fact-laden, sometimes trauma-inducing engagements regarding all the ways systemic racism impacts people of color (125, 127).

Racially conscious training requires intense and skillful conversations about race (128). Critical conversations about race, though necessary for the training, may trigger racial trauma in people of color, whose distress can then trigger interracial anxiety in many White people (e.g., 126, 129, 130). Unfortunately, this being the first event of its kind, MAPS staff were unprepared for the emergence of this trauma by the participants at the meeting. The Black psychologist who secured the grant was an experienced diversity trainer, but because the grant funds were awarded to MAPS, she had no decision-making power, control over the agenda, or ability to select senior members of the training team with experience buffering the type of trauma that can be triggered by the material. Instead, her students who were there to be trained were enlisted to help. In addition, the senior BIPOC participants who were present were not utilized to help other participants who were triggered by the material. The few BIPOC members of the MAPS-selected training team were not qualified to do this work and were themselves triggered and unable to support others. Further, as described in an account by MAPS themselves (122), proper care was not taken to protect the identity of former participants in the MAPS studies, one of whom attended the event as a participant but whose study video was shown to the group without consulting her beforehand. It was traumatic for the participant to see herself on screen, and this triggered fears in participants of color reminiscent of research abuses such as the Tuskegee Syphilis Study (15, 122). As a result, the landmark event was stymied, and some participants felt unsafe, with a few leaving before it was over. The training of the therapist cohort was never completed, as such MAPS did not keep its promise to the therapists of color and their funders.

## Bridging the GAP: Solutions for Better BIPOC Therapy

Despite all these missteps, there is much to be learned from past events. In that respect, MAPS has an opportunity as an institution to improve on mistakes by creating a more positive and inclusive clinical pathway where BIPOC can thrive in their psychedelic training programs. Facilitators trained by MAPS, however, cannot be the sole solution to the dearth of Black clinicians. This burdens training and certification programs with a tremendous bottleneck, especially as Black clinicians are realistically more fearful of the legal and reputational impacts of trying illicit substances. The current US psychology workforce is only 4% Black (131). Of the approximately 6,000 doctoral graduates of clinical psychology programs in the USA each year, about half will become active in client care and approximately 10% (or about three hundred) of these providers are Black (26). But any licensed professional can be trained in the area of psychedelic-assisted therapy. This list includes licensed clinical social worker (LCSW), physician associate (PA), nurse practitioner (NP), medical doctor, licensed professional counselor (LPC), and more.

In attempts to find additional solutions to the lack of appropriately trained providers, the California Institute of Integral Studies (CIIS) operates one of the few academic programs dedicated to educating the next generation of psychedelic practitioners. The CIIS has developed a Certificate in Psychedelic-Assisted Therapies & Research (CPTR) program, which aims to educate and prepare a broad range of mental health professionals for work in the field. The CPTR program is a hybrid of online learning and in-person training retreats that has been occurring for the last 5 years. Scholarships and need-based aid have been established for individuals from underrepresented communities as an opportunity to promote equity within the CPTR program. Though there are efforts to create BIPOC and LGBTQIA+ diversity and representation among CPTR trainees, the majority of the staff and faculty remain White. This only further reinforces the field-wide standard of lacking BIPOC faculty and supervisors (e.g., 10).

It is increasingly evident that more creative suggestions should be undertaken to ensure a more rapid implementation timeline for psychedelics training. Pairing such training programs with psychology departments at Historically Black Colleges and Universities (HBCUs) could bode for one option. Trained culturally competent clinical psychologists are graduating from these institutions and could be recruited to help alleviate the current shortage. The authors strongly recommend academic centers at Predominately White Institutions (PWIs) with newly established psychedelic programs reach out to these HBCUs to initiate an overdue process of cooperation that would mutually benefit both programs. HBCUs, who typically train the majority of black psychologists, would gain access to and training in psychedelic knowledge. Meanwhile, the collaboration would expose typically white students from psychedelic research programs to more BIPOC in their field, and if done correctly, could potentially allow for cross-cultural, inclusive training. In all, the issue of culturally competent therapy is one which must be addressed, in part, by University training programs for psychedelic medicine (Table 2).

**Table 2:** Academic potential in psychedelics.

**Academic centers with psychedelic research programs: USA and Canada**		**Historically black US colleges and universities with psychology programs**	
Yale University	Department of Psychiatry	Howard University	PhD and Clinical and Counseling Psychology Programs
John Hopkins University	Center for Psychedelic & Consciousness Research	Norfolk State University	PhD and Clinical Psychology Program
Massachusetts General Hospital	Center for the Neuroscience of Psychedelics	Jackson State University	PhD and Clinical Psychology Program
University of California, Berkeley	Center for the Science of Psychedelics	Prairie View A&M University	PhD and Clinical Adolescent Psychology Program
Medical University of South Carolina	Psychedelics Research Center	Alabama A&M University	Master of Science in Counseling Psychology with a concentration in clinical psychology
Stanford University	Stanford Psychedelic Science Group	Fisk University	Master of Arts in Psychology with a concentration in clinical psychology
California Institute of Integral Studies	Center for Psychedelics Therapies and Research	North Carolina Central University	Master of Arts in Psychology with a concentration in clinical psychology
University of Wisconsin Madison	Psychoactive Pharmaceutical Investigation, MS	Texas Southern University	Master of Arts in Psychology with a concentration in community-clinical psychology
University of Toronto	Psychedelic Studies Research Program	Virginia State University	Master of Science in Psychology with a concentration in clinical psychology
University of Ottawa	Psychedelics and Spirituality Studies Initiative	Bowie State University	Master of Arts in Counseling Psychology

It is imperative to note that many practicing psychologists (both BIPOC and non-BIPOC) have not likely received adequate graduate level training in cultural competency (136, 137). Just as many medical schools have begun to include specific training in social medicine, it would behoove practitioners to introduce a more effective culturally sensitive, inclusion therapy in the training of new clinicians (138). This would go a long way in raising all aspects of mental health and therapeutic care above and beyond psychedelic-assisted therapy.

Although the path to greater empathy and cultural competency for therapists who want to undertake this kind of work may seem long, there are tools available for continuing education and self-assessment. Use of these tools are necessary to help prevent any harm to BIPOC in the therapeutic setting. We have provided a resource table (Table 3) for both therapists and clients to further educate themselves with opportunities for self-assessment.

**Table 3:** Resource table.

**Institute**	**Location**	**Type of therapy**	**Director**
**Racial justice oriented MDMA / Psychedelic clinics**			
The Sage Institute	San Francisco	Ketamine-Assisted Therapy	Dr. Genesee Herzberg
Sound Mind Center	Philadelphia	Expanded MDMA Access	Dr. Hannah McLane
Behavioral Wellness Clinic	Connecticut	Expanded MDMA Access Ketamine-Assisted Therapy	Prof. Monnica Williams
Sana Healing Collective	Chicago	Ketamine-Assisted Therapy	Meghan Kennedy
**Purpose**	**Topic**	**References**	
**Recommended literature**			
Building Empathy for BIPOC	Addressing the Gap between Action and Intent	Williams et al. (132)	
	Civil Courage and Allyship	(133)	
Assessing Cultural Competency	How to be an anti-racist therapist	(134)	
	Microaggressions	(32)	
	Psychedelic Culture	(135)	

## Building Empathy Toward BIPOC Community: Organizations Centered on BIPOC Voices

There are organizations promoting Black people in psychedelics, including Chacruna's Racial Equity & Access Committee, The Ancestor Project, and People of Color Psychedelic Collective who are committed to bringing BIPOC agency and liberation to the forefront of the conversation. They seek to revolutionize psychedelic culture by making psychedelics more accessible to people of color while also combating the harms produced by the drug war and oppressive systems of injustice. To accomplish this, they are conducting community outreach to educate BIPOC about psychedelics' therapeutic and spiritual foundations, harm reduction, and general drug policy. By building inclusive spaces that allow BIPOC to safely explore psychedelics, they foster an environment that encourages vulnerability and collective healing. These organizations put BIPOC contributions to the field of psychedelics in the spotlight and hope to promote a narrative by and for communities of color. As the decriminalization and legalization of psychedelics becomes more commonplace, dedicated spaces that uplift and prioritize BIPOC voices and well-being are paramount to the evolution of the field of psychedelics.

The next phase of the psychedelic-assisted arm of this work is the *Expanded Access Program*, spearheaded by MAPS, which is now starting at multiple sites throughout the US. Expanded Access, also called Compassionate Use, allows patients to have use of an investigational medical product (one that has not yet been approved by the FDA) outside of a randomized clinical trial. The program's purpose is to grant access to potentially beneficial investigational treatments for people facing a serious or immediately life-threatening condition for which there is no satisfactory treatment currently available. This program will allow MDMA-assisted therapy to be available for people who have not responded to traditional therapies for PTSD, and it will also provide additional data on drug safety and how various subpopulations may respond to the treatment.

There are several sites in the US (Table 3) which are specifically focused on providing this expanded access care for people of color. These sites include *Sage Institute*, a center in California that has applied to be approved for expanded access and is specifically dedicated to providing high-quality, culturally responsive psychedelic therapy for underserved communities. The Sage Institute is active as both a therapeutic investigative site in San Francisco, and the colleagues at Sage have experience in ketamine-assisted therapy. *SoundMind Center* in Philadelphia, also on the list for expanded MDMA access, is a collective of psychiatrists, psychotherapists, and community organizers among others, dedicated to providing affordable, accessible, inclusive mental health care to Philadelphians, especially those who typically face marginalization within the healthcare system. The SoundMind collective currently has nine providers enrolled in FDA-approved training for MDMA assisted therapy and four others already certified in ketamine-assisted therapy. *Behavioral Wellness Clinic* in Connecticut, whose clinic personnel participated in the FDA-approved clinical trial of MDMA-assisted psychotherapy for PTSD, has a diverse FAP-trained and culturally-informed staff that includes seven members already trained on the FDA-approved MAPS protocol and are enrolled for final certification. The clinic also offers ketamine-assisted therapy for several indications, including racial trauma (76). These three sites will be the first specifically qualified to offer MDMA for patients suffering from racial trauma or PTSD caused by racial trauma.

## Conclusion

Racially induced trauma and PTSD caused by racism are serious and widespread psychological issues that remain undertreated and under-researched (11). The body of literature, as reviewed in this paper, shows that people racialized as Black endure discrimination. And, in fact, Black individuals who suffer from racial trauma populate all areas of society regardless of socioeconomic status and educational attainment (77). These individuals are living with a heightened state of fear and trepidation, which has life-long repercussions (11). Such exposure to everyday racism takes its toll, leaving Black people vulnerable to a spectrum of diseases of the brain, mind, and body (139). In the wake of this daily systemic racism is a trail of inadequately treated patients with altered brain connectivity (140). The lack of treatment opportunities is a result of living in a racist society where BIPOC have been historically marginalized. This problem is multifaceted, multilayered and is emblematic of the general failings of American institutions—political, economic, education, healthcare, and justice—to make good on their promises of equality and inclusivity (e.g., higher education, 83, 103) (83). Despite these gross institutional injustices, there are solutions. Psychedelic-assisted treatment has great promise to provide relief for those suffering from PTSD and racial trauma (141). However, these new opportunities risk causing more harm to Black people than healing if personnel are not properly vetted and trained in both psychedelic therapy and race-based trauma. Although some universities are offering isolated courses, there is not a single university psychology or medical-based program offering a specialization in therapy for racial trauma (44). Thus, the current sites that are using culturally informed therapeutic practices need to be bolstered, and training programs with specific expertise in this area require accelerated funding.

Important measures have been assembled that allow for a more diverse recruitment of patients and culturally trained staff with the provision of functional ethical guidelines. However, there are still unmet needs in communities (military, BIPOC) suffering from PTSD and racial trauma (98). We, therefore, call for more explicit invitations for BIPOC medical professionals and psychology departments at HBCUs to become part of the conversation around the testing, prescription, therapeutic administration and commercialization of these medicines as they move into mainstream mental health care. Healing and well-being of the BIPOC community is dependent on such collaborations.

## Data Availability Statement

The original contributions presented in the study are included in the article/supplementary material, further inquiries can be directed to the corresponding author.

## Author Contributions

DS worked on the initial draft, wrote a substantial portion of the sections, invited additional researchers, did the final edits for the paper, invited SF to help organize the sections, and invited LG to write about a section in the paper to include handling the conclusion and references. DF was invited to contribute regarding the brain and psychological impact of race-based trauma. NB wrote about race relations and its implications regarding racial trauma and psychedelic work. All authors contributed to the article and approved the submitted version.

## Conflict of Interest

SF was employed by the company Bioville GmbH. The remaining authors declare that the research was conducted in the absence of any commercial or financial relationships that could be construed as a potential conflict of interest.

## Publisher's Note

All claims expressed in this article are solely those of the authors and do not necessarily represent those of their affiliated organizations, or those of the publisher, the editors and the reviewers. Any product that may be evaluated in this article, or claim that may be made by its manufacturer, is not guaranteed or endorsed by the publisher.

## Footnotes

^1^Black here refers to a “racial group” defined by the US government census. A racial group is often related to, but not equivalent to one's ethnic group identification and is not necessarily associated with genetic relatedness ((12)). For the purpose of this article, Black includes individuals racialized as Black (e.g., Black American, African American, or Black Canadian). These individuals may be native to or trace their origins to countries within the African Diaspora and where the native people typically share darker skin shades; however, settler-colonialism has resulted in Black people now having a full spectrum of skin shades. Importantly, Black is a social category, and as such, a person racialized as Black in the US or Canada may not be considered Black in other nations or societies (13).

^2^It is important to understand “Whiteness” as a construct and not a skin color. Whiteness describes a privileged social and cultural group that acts as the accepted and elevated standard to which other racial groups are compared. It is a forced group membership that originated by oppressing people of color.

^3^This same researcher is also developing a case study in which ketamine-assisted neurofeedback therapy is utilized in a transracial adoptee suffering race-based and abandonment trauma ((77, 78)).

^4^More alarmingly, White veterans with misconduct separations were more likely to receive these disability awards than their Black counterparts who had no negative marks.
